# An interactive task-based method for the avoidance of metal artifacts in CBCT

**DOI:** 10.1007/s11548-024-03103-4

**Published:** 2024-05-23

**Authors:** Maximilian Rohleder, Mareike Thies, Sophie Riedl, Benno Bullert, Jula Gierse, Maxim Privalov, Eric Mandelka, Sven Vetter, Andreas Maier, Bjoern Kreher

**Affiliations:** 1 Pattern Recognition Lab, Friedrich-Alexander-University, Martenstraße 3, Erlangen, 91058 Germany; 2 Siemens Healthineers AG, Siemensstraße 1, Forchheim, 91301 Germany; 3 Department for Trauma and Orthopaedic Surgery, BG Klinik Ludwigshafen, Ludwig-Guttmann-Straße 13, Ludwigshafen am Rhein, 67071 Germany

**Keywords:** Metal Artifact Avoidance, Cone-beam CT, CT Trajectory Optimization, Human Computer Interaction

## Abstract

**Purpose:**

Intraoperative cone-beam CT imaging enables 3D validation of implant positioning and fracture reduction for orthopedic and trauma surgeries. However, the emergence of metal artifacts, especially in the vicinity of metallic objects, severely degrades the clinical value of the imaging modality. In previous works, metal artifact avoidance (MAA) methods have been shown to reduce metal artifacts by adapting the scanning trajectory. Yet, these methods fail to translate to clinical practice due to remaining methodological constraints and missing workflow integration.

**Methods:**

In this work, we propose a method to compute the spatial distribution and calibrated strengths of expected artifacts for a given tilted circular trajectory. By visualizing this as an overlay changing with the C-Arm’s tilt, we enable the clinician to interactively choose an optimal trajectory while factoring in the procedural context and clinical task. We then evaluate this method in a realistic human cadaver study and compare the achieved image quality to acquisitions optimized using global metrics.

**Results:**

We assess the effectiveness of the compared methods by evaluation of image quality gradings of depicted pedicle screws. We find that both global metrics as well as the proposed visualization of artifact distribution enable a drastic improvement compared to standard non-tilted scans. Furthermore, the novel interactive visualization yields a significant improvement in subjective image quality compared to the state-of-the-art global metrics. Additionally we show that by formulating an imaging task, the proposed method allows to selectively optimize image quality and avoid artifacts in the region of interest.

**Conclusion:**

We propose a method to spatially resolve predicted artifacts and provide a calibrated measure for artifact strength grading. This interactive MAA method proved practical and effective in reducing metal artifacts in the conducted cadaver study. We believe this study serves as a crucial step toward clinical application of an MAA system to improve image quality and enhance the clinical validation of implant placement.

## Introduction

Mobile C-Arm systems are used for guidance using 2D imaging during orthopedic and trauma procedures and are increasingly used for 3D verification of implant placement using cone-beam CT (CBCT) capabilities. Especially in treatment of spinal fractures, the intraoperative validation of correct implant positioning using 3D imaging is crucial, as the anatomies of interest and placement of screws within them is hard to verify on projection images. In this context, metal artifacts emerging in and around metallic objects in the CBCT reconstruction obstruct clinically relevant image features and therefore harden clinical decision making.

The field of trajectory optimization has investigated several approaches to improving the image quality by adapting the scanner trajectory which are summarized in the review paper by Hatamikia et al. [3]. General implant-agnostic orbits have been investigated and several non-circular sampling strategies have been shown to reduce metal artifacts in general [2]. To customize the trajectory to the object to be imaged, approaches have been suggested which employ prior knowledge of the scene such as a full 3D model or a pre-op CT [3]. With trauma applications in mind, where a pre-op CT might be outdated, an approach investigates the feasibility of optimizing a circular or non-circular trajectory given a few scout view X-ray images [10]. Instead of prior knowledge, one approach investigates the online adaptation of the scanner tilt based on the current projection images during the 3D scan [8]. Related work also investigates theories on task-driven optimization of source–detector trajectories to tailor the imaging trajectory optimally for a given well-formulated imaging task [7].

In this work, we build on the scout view-based approach by Wu et al. [10] and address remaining challenges currently preventing the clinical application of this method. While previous studies have demonstrated the improved image quality achievable with non-circular trajectories, we have chosen to exclusively employ tilted circular trajectories. This decision is motivated by our commitment to prioritize clinical translation, considering the practical and regulatory constraints associated with non-circular acquisitions. Especially in the context of spine surgery, it has been shown that changing the acquisition plane of a circular scan has a significant effect on subjective image quality and clinical assessability [4].

We propose an interactive optimization scheme to be integrated with the current imaging workflow as user-in-the-loop optimization. To enable the clinician to find a more suitable trajectory, we present a method for localization of expected artifacts within the volume of interest and visualize this information as an interactive overlay onto the scout views. Using the proposed colored overlay as guidance, we seek to enable the clinician to find an optimized scanning trajectory while factoring in an imaging task derived from clinical conditions and the procedural context. A side effect of spatially localized artifact metrics is that we are able to propose a protocol to calibrate the computed metrics to observed artifact strengths. Thus, the predictive metrics expressed through colors in the guidance overlay allow to conclude absolute strength of artifacts to be expected.

We summarize the following contributions:

- We propose an integration of the MAA system into the imaging workflow as human-in-the-loop optimization and test its feasibility and efficacy in a cadaver study.
- We detail a method to predict the expected absolute strength and 3D localization of metal artifacts from given scout X-ray images and visualize this information interactively to guide trajectory optimization.
- We show that the performance of circular orbit optimization can be improved by incorporating an imaging task using this method.

## Methods

### Angular and spatial metal artifact localization

**Fig. 1:**
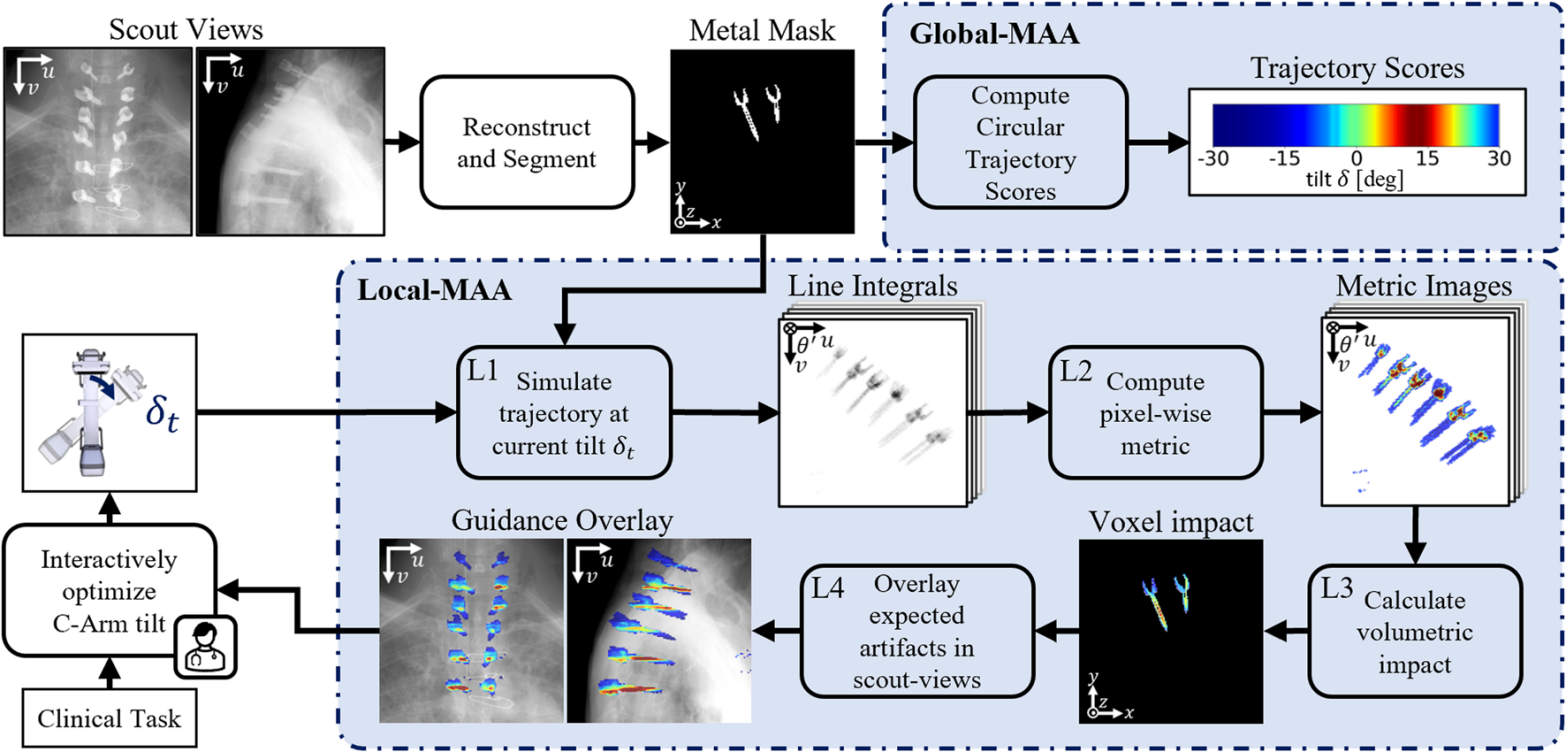
Method for localization of predicted metal artifacts and interactive circular orbit optimization workflow. First, a 3D metal mask is reconstructed from given scout view X-ray images. To calculate **Global-MAA** scores, the variation of spectral shift is computed similar to [10] (top right). To compute the **Local-MAA** guidance overlay, line integrals are computed at the current gantry tilt (L1) and mapped to model the metal induced bias (L2). The volumetric distribution of expected artifacts is then computed within the metal mask by backprojection and variance weighting (L3). By maximum projection onto the scout views, an overlay images is generated (L4). Based on this guidance, the user interactively chooses a trajectory by tilting the C-Arm to minimize the displayed artifact prediction

The processing pipeline shown in Fig. 1 is employed to calculate the Global-MAA scores (top right) and spatially resolved Local-MAA overlays for the current C-Arm tilt (bottom). Given two or more scout views of the scene, the metal distribution is estimated as a volumetric metal mask $b_{seg}(x, y, z)$. This can be done either by backprojection and subsequent segmentation of metallic objects as detailed in Wu et al. [10] or using a end-to-end deep learning approach as described in Rohleder et al. [5]. This component of the pipeline is assumed given and is therefore excluded from investigations in this work.

From this knowledge of metal distribution, a score is derived for each trajectory following the $Q_{poly}$ objective function from Wu et al. [10]. Projection images are computed for each tilted circular scan and the mean spectral shift per simulated projection is computed. A trajectory is scored by computing the variance over the averaged spectral shift values of its constituting projection images. The resulting 1D objective function $Q_{poly}(\delta )$ is normalized relative to the worst and best possible trajectory over the evaluated angular range from $\delta \in [-30, 30]$ degrees. Details for this procedure can be found in [10].

To compute the spatial distribution of expected metal artifacts (Local-MAA, bottom part of Fig. 1) we follow a slightly differing approach. Initially, the current C-Arm tilt $\delta _t$ is read out and path length images $p_{\delta _t}(u, v, \theta )$ are computed as line integrals through the binary metal volume $b_{seg}(x, y, z)$ (L1). Defining the system matrix $\mathbf {A_{\theta , \delta _t}}$ as the Siddon raytracing forward projection [6] for a given C-Arm tilt $\delta _t$ and rotation $\theta$, this can be expressed as

1 $$\begin{aligned} p_{\delta _t}(u, v, \theta ) = \textbf{A}_{\theta , \delta _t}b_{seg}. \end{aligned}$$

To better model the artifact bias induced by a certain projection length of metal, we compute the spectral shift defined as the difference between the monoenergetic and polyenergetic forward model similar to Wu et al. [10] (L2). However, in contrast to the Global-MAA computation, we compute this per pixel instead of summing over each projection image.

In step L3, the volumetric impact per unit volume of these projection domain spectral shift maps is calculated. For each voxel defined as metal in the metal mask $b_{seg}$ a value is computed as the variance over its projected locations’ values in the previously computed spectral shift images. While this seems similar to the objective function $Q_{poly}$ the notable difference here is that this value is normalized over a unit volume and is thus an absolute measure for the expected artifact strength at this volumetric location. This results in the voxel impact maps $m_{\delta _t}(x, y, z)$ which can be expressed as

2 $$\begin{aligned} m_{\delta _t}(x,y,z) = \textrm{Var}_\theta (s_{\delta _t}(u^*, v^*, \theta )), \end{aligned}$$

where $u^*$ and $v^*$ are the coordinates of position (*x*, *y*, *z*) after forward projection into view $\theta$ under tilt angle $\delta _t$.

**Fig. 2:**
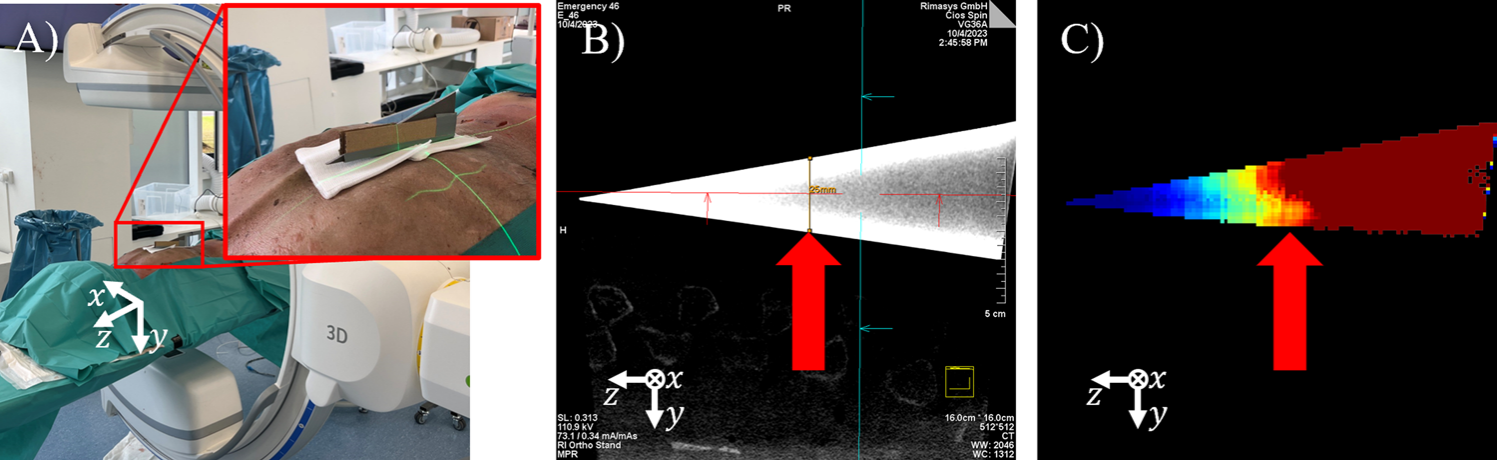
Overlay color calibration protocol. A calibration phantom (titanium wedge) is scanned as depicted in A). The resulting CBCT volume is inspected and an axial slice is determined where the observed metal artifacts are intolerable (see red arrow in B). Steps L1–L3 from the method described in Fig. 1 are conducted and the same point is determined in the voxel impact map in C). The average value at this position is used to define the color mapping illustrated in C)

Finally, in step L4 the overlay images are computed by the maximum projection of the voxel impact map onto the two scout views along the known projection geometry.

**Implementation Details** To facilitate real-time updates of the rendered overlay images, steps L1–L4 are implemented as CUDA kernels. The variance is calculated efficiently using Welford’s online algorithm [9]. Typical resolutions of the volumetric metal mask and the voxel impact maps are $128^3$ at a isotropic resolution of 1.2mm per voxel side. A trajectory is simulated as 200 images over 200 degrees rotation at a resolution of $244^2$ pixels which corresponds to 1.3mm physical pixel size. To enable an interactive exploration of the artifact distribution, a prototype is developed which integrates with the C-Arm to read out angle encoder values.

### Color calibration for visualization

In order to standardize the unbounded and initially incomprehensible values derived from the variance of spectral shift per voxel unit volume, a basic calibration method was developed. The objective is to establish a metric value that represents a significant degree of artifacts and designate this value as the upper limit in the color map used for presentation to the physician. As illustrated in Fig. 2, a wedge phantom (50x150x5mm) made of titanium is positioned along the z-axis of the C-Arm gantry on top of a cadaver for realistic patient contrast. After acquisition of a CBCT scan, the volume shown in Fig. 2B is inspected in axial slices for empirically identifying a “critical artifact threshold.” In the determined slice, the height of the wedge phantom was measured as 25 mm. This was done by a trained orthopedic surgeon by evaluating the homogeneity of intensity within the metallic object and the severity of streaking artifacts surrounding the wedge phantom. Using a threshold-based segmentation of the object, steps L1–L3 of the previously described method are evaluated to yield the voxel impact map. The mean value at the empirically determined height is read out and henceforth used to normalize the computed voxel impact for color hue mapping in the final overlay images.

We hypothesize that this calibration protocol works, as the phantom models a monotonous increase in variance of spectral shift. The thickness of the wedge is loosely similar to the diameter of pedicle screws (approx. 5 mm) while the increasing height models increasing shaft lengths.

### Interactive trajectory optimization using localized metal artifact predictions

Based on the dynamically changing computed Local-MAA guidance, we propose an interactive optimization scheme. As illustrated in Fig. 1, the clinician interprets the displayed overlay and adjusts the C-Arm tilt, which in turn changes the shown guidance image. This interactive setting has several advantages. Making use of the clinical background knowledge and procedural context allows to condition the tilt optimization problem and find a imaging task-specific optimum. Furthermore, should practical considerations such as obstructions in the OR interfere with an optimal circular orbit, this can be accounted for and a next-best trajectory can be found as compromise. Finally we hypothesize the absolute localized metrics allow to judge if a tilt is necessary at all, which is not possible with Global-MAA scores as these are always normalized to best/worst case. The envisioned workflow is further demonstrated in the supplementary material of this paper.

## Experiments

This study aims to evaluate the interactive MAA workflow within a realistic setting, while assessing the image quality of the Local-MAA against the Global-MAA and scans without any MAA system. Evaluation will be conducted both across the entire image and within a defined task region of interest. Additionally, we seek to validate the proposed color calibration method crucial for absolute artifact strength grading.

### Cadaver study design

To realistically assess the efficacy of the proposed approach, a cadaver study is conducted. Three cadavers of varying body mass index $[22.66, 26.38, 33.98\text {kg}/\text {m}^{2}]$ are selected and a dorsal instrumentation within the lumbar and thoracic region of the spine with pedicle screws is applied by a trained trauma surgeon (“the user”). This results in a total of six “scenes” defined as unique combinations of cadaver region and instrumentation.

To be able to reconstruct CBCT volumes from tilted circular source–detector orbits, a series of scans of a geometric calibration phantom are acquired using a Siemens Cios Spin Mobile C-Arm system. In total, 13 scans ranging from -30 to 30 degrees in 5 degree steps are collected. From these scans, projection matrices are calculated following the method from Cho et al. [1]. For a new tilted acquisition, the device’s tilt angle encoder is read out with 0.1^∘^ precision and projection matrices are calculated from the neighboring calibration matrices via interpolation of C-Arm pose and imaging intrinsics like in [10].

### Experiment 1: Assessment of image quality improvements

In the first experiment, the effectiveness of the proposed approach was evaluated through a comprehensive assessment of image quality. This assessment involved several steps. Initially, 3D scans were acquired for each of the six distinct scenes, with subsequent segmentation of metallic objects using a pre-trained UNet. In this study, we opted to use the full reconstruction rather than scout images to compute the metal mask, ensuring that we exclusively assess the impacts of the methods under evaluation. This approach minimizes the potential for errors resulting from inaccurate segmentation. The 3D metal masks derived from this segmentation were visually checked for completeness and were then employed to compute both global trajectory scores and localized MAA overlay guidance views, as specified in Sect. 2.1.

To further evaluate the proposed method’s practical utility, specific tasks were defined for each scene based on the anatomical and surgical context. Typically, the vertebra with the smallest pedicle, known to be challenging for screw placement, was selected. Then, an optimal C-Arm tilt was determined by the user, guided separately by the Local-MAA overlay images and the 1D metric derived from the Global-MAA method. As a reference point, a non-tilted standard trajectory was used.

The resulting image quality was assessed by three trained trauma surgeons, each individually evaluating every pedicle screw on a 5-point Likert scale. The assessment criteria encompassed the clarity of screw form, clarity of the surrounding tissue and the overall subjective image quality, all within the context of intraoperative 3D imaging for pedicle screw placement validation. Notably, to ensure an unbiased evaluation process, a clinically utilized 3D image viewer presented the image data in a non-labeled and non-ordered format.

This process resulted in the labeling of 96 screws per surgeon (32 screws imaged using each of the three compared methods), contributing to a total dataset of $N=288$ data points for the quantitative analysis of the proposed method’s efficacy and comparison to the two reference methods.

### Experiment 2: Evaluation of overlay color calibration

Additionally, a second experiment was conducted to validate whether the calibrated predicted artifact magnitude conveyed through the overlay colors coincides with quantifiable image artifacts after reconstruction. A prevalent phenomenon associated with metal artifacts is the so called blooming artifact. This term describes the effect that metallic objects, i.e., a pedicle screw appears to have a increased diameter (“it blooms”) in the reconstructed image.

The experimental procedure begins with the execution of the color calibration process on the cadaver with the largest BMI, adhering to the protocol outlined in Sect. 2.2. Subsequently, eight CBCT scans of two pedicle screws inserted in the lumbar spine of the medium-sized cadaver are acquired covering a tilt range from $-15^\circ$ to $20^\circ$ in $5^\circ$ steps. After image reconstruction, the CBCT volumes were automatically analyzed, involving the following steps: The eight CBCT volumes were co-registered, the screw shaft was manually annotated by selecting the head and tip, and a series of measurements were conducted at the center of the screw shaft. Specifically, measurements were taken at 7 levels along the screw shaft center spaced 0.5mm apart and 18 rays in 10-degree increments rotated around the screw axis, measuring the thickness defined by full width at half maximum (FWHM) as a standardized measuring protocol. A total of 126 thickness measurements were collected per screw and subsequently summarized in terms of mean and standard deviation.

To validate the calibration process, steps L1–L3 of the Local-MAA pipeline, as described in Sect. 2.1, were computed for each angle at which a CBCT volume was acquired. Finally, the mean calibrated color within the region of interest (ROI) around the center of the screw shaft was compared against the observed quantified blooming artifact.

## Results

### Comparison of image quality improvements

**Fig. 3:**
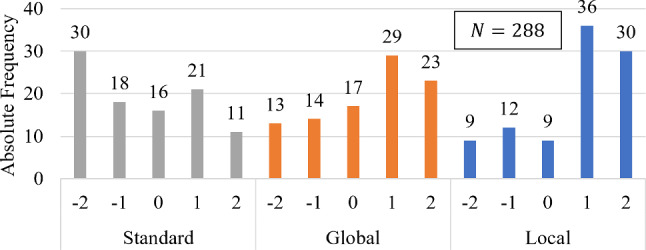
Subjective image quality of 96 pedicle screw images graded by three practicing orthopedic surgeons on a scale from $[-2, 2]$. The CBCT scans guided by local artifact visualization (Local-MAA) are compared to trajectory averaged scores (Global-MAA) as tilt selection guidance and standard non-tilted scans (No-MAA)

**Fig. 4:**
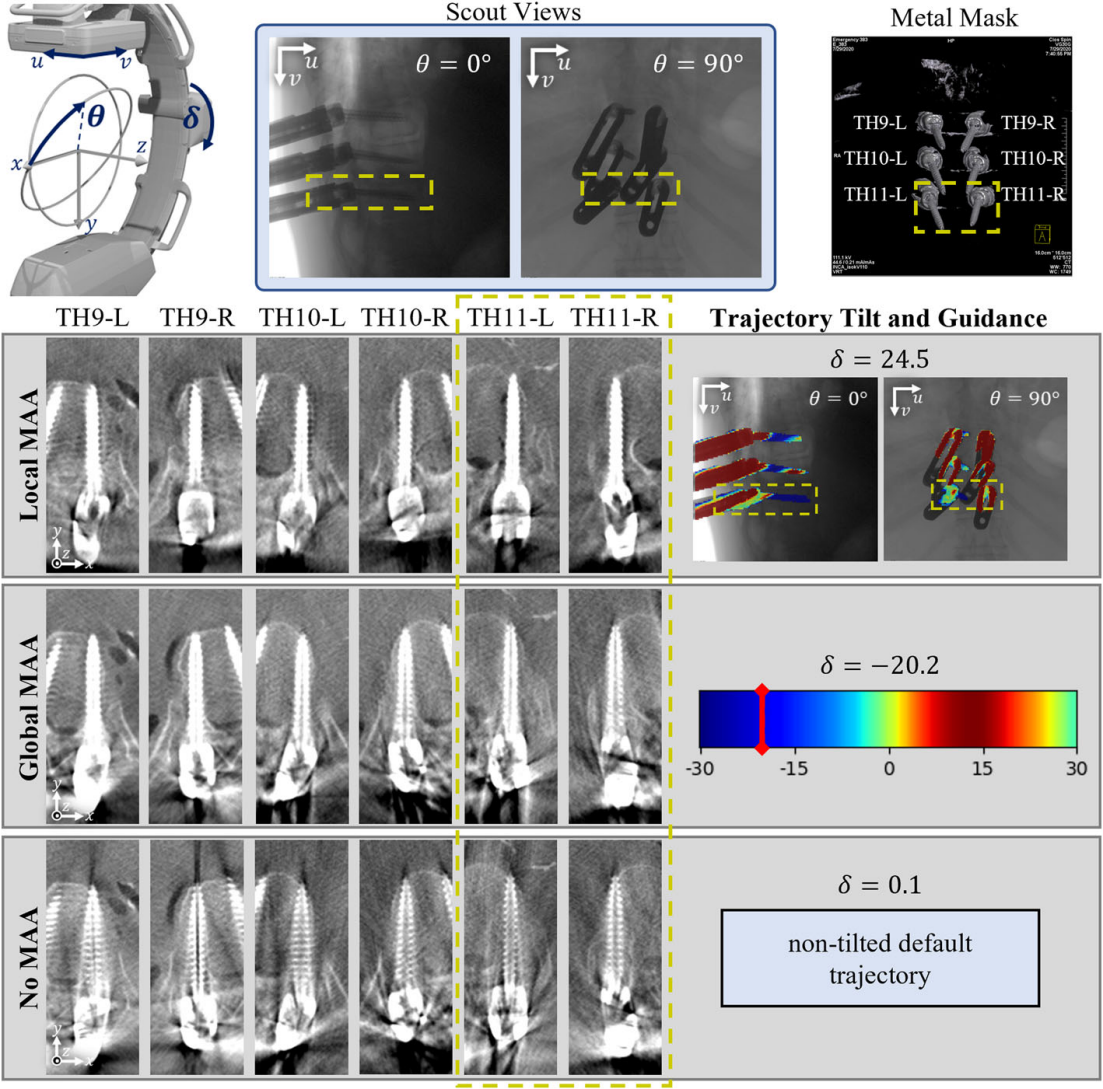
Visual comparison of resulting image quality using Local-MAA interactive optimization with defined task on thoracic vertebra TH11, global trajectory scores (Global-MAA) and without any MAA system (No-MAA). The six pedicle screws in vertebrae TH9–TH11 are depicted alongside the guidance overlay and trajectory score map used to decide for a C-Arm tilt

The aggregated subjective image quality grades ($N=288$) of the pedicle screw images are presented in Fig. 3. Comparing the distribution of grades between the evaluated methods, it becomes evident that both Global- and Local-MAA lead to visually better graded pedicle screw depictions compared to using no MAA system.

The Wilcoxon signed-rank test (from the SciPy library) was additionally used to compare the pair-wise, ordinal-scaled, non-normally distributed gradings, testing the null hypothesis that there is no central tendency between the gradings of the three methods. The results indicated significant differences. The Local-MAA method demonstrated notably better image quality compared to the Global-MAA method (W=455.5, p=0.0305) and the non-tilted standard scans (W=565.0, p=4.93e$-$07). Similarly, the Global-MAA method outperformed the standard reference scans (W=343.0, p=6.59e$-$08) in subjective image quality.

In addition to these quantitative findings, Fig. 4 illustrates an example. The scene includes six pedicle screws and towers in vertebra TH9 through TH11 of the thoracic spine. As an imaging task, the pedicle screws in vertebra TH11 are selected from procedural context. The guidance image produced by the proposed Local-MAA method for the selected tilt of positive 24.5^∘^ shows the screws in TH11 (top) in blue color. The trajectory scores from the globally averaged MAA method on the other hand suggest to tilt into negative direction. Comparing the depicted pedicle screws, it is obvious that both tilted acquisitions drastically improve image quality compared to the bottom row featuring the reference scan. However, looking at the right two columns, we observe that the scan acquired with Local-MAA optimizes for the screws in TH11, whereas the global MAA optimizes for global image quality including the towers or other obstacles, which are not relevant in this use case.

#### Influence of task formulation on Local-MAA

**Fig. 5:**
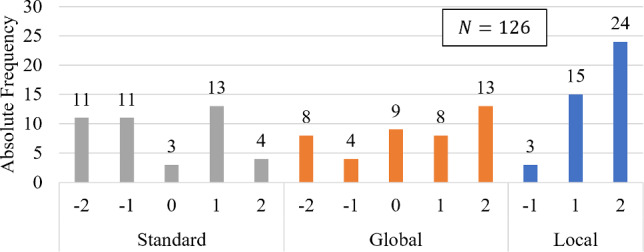
The subset of 42 grades related to screws which are defined as the imaging task due to clinical context are compared between the three evaluated methods

Secondly we selectively compare pedicle screws which were defined as an imaging task during the study. Figure 5 shows the updated distribution of the remaining $N=126$ grades. The gradings originating from scans chosen using the Local-MAA system received almost only positive gratings. In comparison, both Global-MAA and reference scans received far more negative gradings.

**Fig. 6:**
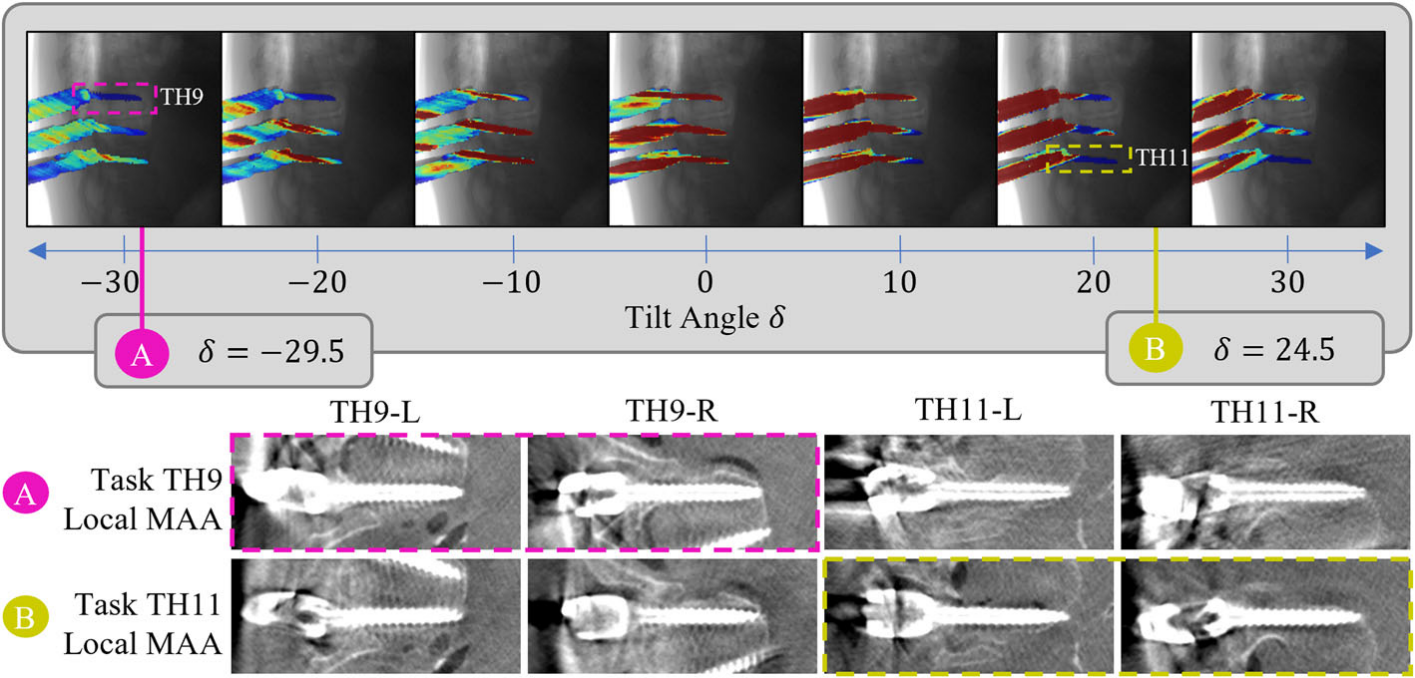
Illustration of task-selective circular orbit selection. Depending on the clinical and procedural context the pedicle screws in either thoracic vertebra TH9 or TH11 are selected which leads to changes in selected tilt angle and metal artifact distribution

The influence of task choice on selected tilt and artifacts is illustrated in Fig. 6. Extending the example from Fig. 4, a second task is formulated to showcase the change in selected trajectory and resulting artifact distribution. The formulation of TH9 as imaging task results in a negative tilt which is drastically different than for the previous task. Looking at the resulting images, it can be validated that the image quality indeed increases as the screw shaft is depicted more homogeneous. However, the differences are not as pronounced as comparing to the standard trajectory in this case.

### Correspondence of displayed color and measured artifacts

**Fig. 7:**
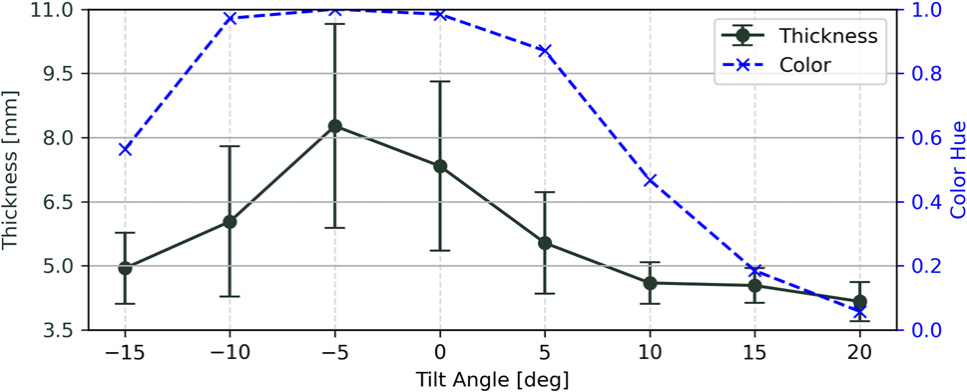
The blooming artifact of a 4.5mm diameter screw is measured as increase of mean thickness and standard deviation. This is compared to the predicted metal artifact severity expressed through voxel impact metrics mapped to a color hue through the proposed color calibration protocol

To assess the predictive quality of the modeled voxel-level variance of spectral shift, Fig. 7 shows the measured blooming artifact and calibrated color over a series of tilted acquisitions. The shape of predicted artifacts and measured blooming are in good visual correspondence which indicates a good agreement between predicted and observed metal artifact severity.

## Discussion and Conclusion

This work introduces a novel scout view-based approach toward MAA using tilted circular orbit optimization. This approach not only enables the localization and visualization of anticipated metal artifacts but also provides a viable interactive workflow integration and a means to estimate the absolute artifact strength, setting it apart from previous techniques. Our systematic evaluation, conducted in a realistic cadaver study, demonstrated the efficacy and practicality of our proposed Local-MAA system. The significant improvements in subjective image quality, particularly when compared to non-tilted CBCT acquisitions and scans derived from the 1D trajectory scores computed by the Global-MAA, highlight the potential clinical impact of our method.

Importantly, our findings underscore the value of task-based optimization, as evidenced by the influence of imaging tasks on trajectory selection and subsequent artifact reduction demonstrated in results section 4.1. The ability to prioritize specific regions of interest based on clinical tasks bears the potential to have a substantial impact on the image quality of intraoperative validation CBCT scans.

In this work we aim to devise a system with broad practical clinical applicability. This principle motivates two design choices. First, as mentioned in the introduction of this work, we choose circular over non-circular trajectory optimization despite the proven better performance of the latter [10]. This is because obtaining a customized non-circular scan necessitates a fully calibrated robotic C-Arm system and proves challenging from a regulatory standpoint, as a collision check is required prior to motorized data acquisition. Secondly, we investigate human-in-the-loop instead of automated optimization to find the optimal tilted trajectory. While this interactive solution may introduce complexity and the potential for human error in the 3D imaging workflow, it elegantly allows the user to prioritize a clinically relevant region while simultaneously considering the movement constraints imposed by the surrounding operating room scene. While it is technically feasible to condition automatic circular trajectory optimization on a clinically relevant local region (e.g., a specific screw), incorporating the specific movement constraints posed by obstacles in the operating room still presents a challenge. Furthermore, this interactive tilt optimization does not require a fully robotic and calibrated system, which makes it applicable to a wider fleet of C-Arm systems.

A key advantage of our proposed method lies in its volumetric localization of expected artifacts, which directly indicates the artifact strength at the identified locations. While our rudimentary calibration protocol and used metric demonstrates promising agreement, we acknowledge its limited modeling capability for absolute artifacts, particularly in disregarding various contributing factors such as scattered radiation, patient attenuation and device settings. On the same note, a more comprehensive evaluation of the correlation between computed voxel impact metrics and observed artifacts is necessary to establish the robustness of this calibration approach.

Furthermore, it is important to highlight limitations of this study and identify areas for future research. These include the assumptions related to metal segmentation which is for the scope of this study not conducted from scout views but from CBCT reconstructions. Future work should evaluate the full pipeline from scout views to reduced artifacts and assess the influence of segmentation error on subsequent trajectory choices. As this study primarily aims to demonstrate initial feasibility, we intentionally limited the scope to pedicle screw surgery. The decision to involve three trained surgeons who assessed 32 images, resulting in 96 paired samples, was guided by practical considerations inherent in conducting cadaveric studies, including the availability of skilled trauma surgeons, ethical considerations and the resource-intensive nature of such experiments. To comprehensively cover all clinical situations, future work should include an evaluation covering a broader range of implants, procedures, cadavers and users interpreting the displayed overlays. Additionally, a comparison to non-circular trajectories and post-processing Metal Artifact Reduction methods would be of scientific interest.

In conclusion, the proposed interactive MAA method offers a practical and effective approach to avoid the emergence of metal artifacts in clinical CBCT images by means of tilted circular scanning trajectories. The proposed interactive tilt optimization can be elegantly integrated into the 3D imaging workflow between positioning of the C-Arm’s iso-center using two scout views and the collision check. If future work finds the segmentation of metal in 3D from these two X-ray projections sufficient, this approach requires no added dose. In clinical practice, it remains to be seen, if such a system is used by users of varying levels of experience. The results from this study and other experimental studies [4] indicate a significant improvement of image quality with subsequent better assessability of fracture reduction and implant positioning and thus potentially improved quality of care.

## References

[1] Cho Y, Moseley DJ, Siewerdsen JH, Jaffray DA (2005). Accurate technique for complete geometric calibration of cone-beam computed tomography systems. Med Phys.

[2] Gang GJ, Stayman JW (2022). Universal orbit design for metal artifact elimination. Phys Med Biol.

[3] Hatamikia S, Biguri A, Herl G, Kronreif G, Reynolds T, Kettenbach J, Russ T, Tersol A, Maier A, Figl M, Siewerdsen JH, Birkfellner W (2022) Source-detector trajectory optimization in cone-beam computed tomography: a comprehensive review on today’s state-of-the-art. Phys Med Biol **67**(16), 16TR03. 10.1088/1361-6560/ac8590 10.1088/1361-6560/ac8590 35905731

[4] Privalov M, Bullert B, Gierse J, Mandelka E, Vetter SY, Franke J, Grützner PA, Swartman B (2023). Effect of changing the acquisition trajectory of the 3d c-arm (cbct) on image quality in spine surgery: experimental study using an artificial bone model. J Orthop Surg Res.

[5] Rohleder M, Kunze H, Kleinszig G, Maier A, Kreher B (2024) 3d metal segmentation from few x-ray images for metal artifact avoidance. In: Fahrig, R., Sabol, J.M. (eds.) Medical Imaging 2024: Physics of Medical Imaging. vol. 12925. SPIE. 10.1117/12.3005260

[6] Siddon RL (1985). Fast calculation of the exact radiological path for a three-dimensional CT array. Med Phys.

[7] Stayman JW, Capostagno S, Gang GJ, Siewerdsen JH (2019) Task-driven source-detector trajectories in cone-beam computed tomography: I. Theory and methods. J Med Imaging **6**(02), 1. 10.1117/1.jmi.6.2.025002 10.1117/1.JMI.6.2.025002 PMC6497008 31065569

[8] Thies M, Zäch JN, Gao C, Taylor R, Navab N, Maier A, Unberath M (2020) A learning-based method for online adjustment of C-arm Cone-beam CT source trajectories for artifact avoidance. Int J Comput Assist Radiol Surg 15(11):1787–1796. 10.1007/s11548-020-02249-1 10.1007/s11548-020-02249-1 PMC7603453 32840721

[9] Welford BP (1962). Note on a method for calculating corrected sums of squares and products. Technometrics.

[10] Wu P, Sheth N, Sisniega A, Uneri A, Han R, Vijayan R, Vagdargi P, Kreher B, Kunze H, Kleinszig G, Vogt S, Lo SF, Theodore N, Siewerdsen JH(2020) C-arm orbits for metal artifact avoidance (MAA) in cone-beam CT. Phys Med Biol **65**(16). 10.1088/1361-6560/ab9454 10.1088/1361-6560/ab9454 PMC8650760 32428891

